# Supplementary material for: Improving outcomes for donation after circulatory death kidney transplantation: Science of the times
Source: PLoS One. 2020 Jul 29;15(7):e0236662. doi: 10.1371/journal.pone.0236662 (PMC7390443; doi:10.1371/journal.pone.0236662)
Supplement: S1 Table — In total 16 multivariate analyses were performed. Each row represents a single multivariate analysis. The multivariate models were adjusted for variables that were statistically relevant in the univariate analysis (p-value <0.1). (-) Indicates that the variable was not included in the multivariate analysis. (*) p-value <0.05, (**) p-value <0.005. 95% CI, 95% confidence interval; DGF, delayed graft function; eGFR, estimated glomerular filtration rate; HR, hazard ratio; OR, odds ratio. (DOCX) [file pone.0236662.s001.docx]

|  | Donor age | Recipient age | Time on dialysis | Cold ischemic period | Graft anastomosis time |
| --- | --- | --- | --- | --- | --- |
| Delayed graft function  1998-2008 (OR + 95% CI)  2008-2018 (OR + 95% CI) | 1.010 (1.004 - 1.017)**  1.013 (1.007 - 1.020)** | 1.002 (0.995 - 1.008)  - | 1.069 (1.029 - 1.110)**  1.106 (1.065 - 1.148)** | -  1.030 (1.015 - 1.046)** | 1.007 (1.001 - 1.014)*  - |
| Early graft loss (<day 90)  1998-2008 (OR + 95% CI)  2008-2018 (OR + 95% CI) | 1.018 (1.010 - 1.027)**  1.028 (1.014 - 1.041)** | -  1.002 (0.990 - 1.015) | -  1.100 (1.037 - 1.167)** | 1.032 (1.015 - 1.049)**  1.027 (1.001 - 1.053)* | 1.017 (1.009 - 1.024)**  1.018 (1.008 - 1.028)** |
| 1-year eGFR DGF -  1998-2008 (β + 95% CI)  2008-2018 (β + 95% CI) | -0.535 (-0.593 - -0.476)**  -0.512 (-0.577 - -0.448)** | -0.153 (-0.217 - -0.088)**  -0.112 (-0.181 - -0.042)** | -  - | -0.308 (-0.439 - -0.176)**  - | -  - |
| 1-year eGFR DGF +  1998-2008 (β + 95% CI)  2008-2018 (β + 95% CI) | -0.488 (-0.566 - -0.409)**  -0.527 (-0.611 - -0.442)** | -0.061 (-0.145 - 0.022)  -0.006 (-0.093 - 0.080) | -  - | -  - | -  - |
| 1-year graft loss  1998-2008 (HR + 95% CI)  2008-2018 (HR + 95% CI) | 1.021 (1.014 - 1.028)**  1.026 (1.015 - 1.037)** | -  1.001 (0.991 - 1.011) | -  1.080 (1.028 - 1.134)** | 1.029 (1.016 - 1.043)**  1.023 (1.002 - 1.045)* | 1.012 (1.007 - 1.018)**  1.012 (1.004 - 1.021)** |
| 5-year graft loss  1998-2008 (HR + 95% CI)  2008-2018 (HR + 95% CI) | 1.024 (1.018 - 1.030)**  1.018 (1.011 - 1.026)** | 0.986 (0.981 - 0.992)**  - | -  1.043 (1.001 - 1.087)* | 1.022 (1.011 - 1.033)**  1.024 (1.007 - 1.041)* | 1.008 (1.003 - 1.012)**  1.013 (1.007 - 1.020)** |
| 1-year patient survival  1998-2008 (HR + 95% CI)  2008-2018 (HR + 95% CI) | 1.015 (1.005 - 1.026)**  1.006 (0.995 - 1.017) | 1.049 (1.035 - 1.063)**  1.049 (1.034 - 1.064)** | -  - | -  - | -  - |
| 5-year patient survival  1998-2008 (HR + 95% CI)  2008-2018 (HR + 95% CI) | 1.018 (1.011 - 1.024)**  1.011 (1.003 - 1.018)** | 1.049 (1.040 - 1.058)**  1.057 (1.047 - 1.067)** | 1.071 (1.033 - 1.112)**  1.083 (1.044 - 1.123)** | 0.992 (0.978 - 1.006)  1.021 (1.005 - 1.036)* | 1.003 (0.997 - 0.010)  1.012 (1.005 - 1.018)** |

**S1 Table. Multivariate analyses of posttransplant outcomes.**In total 16 multivariate analyses were performed. Each row represents a single multivariate analysis. The multivariate models were adjusted for variables that were statistically relevant in the univariate analysis (p-value <0.1).
(-) Indicates that the variable was not included in the multivariate analysis. (*) p-value <0.05, (**) p-value <0.005.
95% CI, 95% confidence interval; DGF, delayed graft function; eGFR, estimated glomerular filtration rate; HR, hazard ratio; OR, odds ratio.
